# Authentication: A Standard Problem or a Problem of Standards?

**DOI:** 10.1371/journal.pbio.1002477

**Published:** 2016-06-14

**Authors:** Amanda Capes-Davis, Richard M. Neve

**Affiliations:** 1 CellBank Australia—Children’s Medical Research Institute, Westmead, New South Wales, Australia; 2 Gilead Sciences Inc, Foster City, California, United States of America

## Abstract

Reproducibility and transparency in biomedical sciences have been called into question, and scientists have been found wanting as a result. Putting aside deliberate fraud, there is evidence that a major contributor to lack of reproducibility is insufficient quality assurance of reagents used in preclinical research. Cell lines are widely used in biomedical research to understand fundamental biological processes and disease states, yet most researchers do not perform a simple, affordable test to authenticate these key resources. Here, we provide a synopsis of the problems we face and how standards can contribute to an achievable solution.

## Abstract 2

A major contributor to lack of reproducibility in preclinical research is insufficient quality assurance of reagents used. This article examines the problems surrounding the authentication of cell lines and discusses potential solutions.

## Introduction

Statistician George Box famously stated, “all models are wrong.” While referring to mathematical models, there are many parallels from this statement to the biological models used in biomedical research. Whether a biological model relies on cell-free biochemical assays, cell-based assays, or genetically engineered organisms, the validity of the system can, in most cases, be called into question. This is part of the scientific process and should be encouraged.

Blind use of a model system without first conducting fundamental quality controls to ensure its validity should never be tolerated. In the absence of such vetting, validity of results cannot be assessed, resulting in work that at best is impossible to interpret and, at worst, generates meaningless data, use of the same misleading model by others, and wasted research resources. Use of invalid models, such as misidentified (MI) or cross-contaminated (CC) cell lines (Box 1), is an endemic problem within our community that is now widely recognized but not yet widely addressed. Authentication and characterisation of cell lines (Box 2) used by researchers will address this issue. It must be stressed here that ensuring reproducibility does not guarantee that results are correct—it only provides confidence in the measurements, as part of a broader need to generate reliable data with appropriate interpretation [1].

**Box 1. The Problem of Cell Line Misidentification**

Cell lines require constant quality assurance because, as living models, they can change over time. Many cell lines are decades old—sometimes older than the scientists who work with them. Cell lines can change phenotypic behaviour as they continue to be handled. Cell lines can also be contaminated by microorganisms, including bacteria, fungi, and viruses [2]. Whilst any change may affect the cell line’s ability to act as a relevant model, the most radical change is cross-contamination—the quiet substitution of that cell line by another culture. Cross-contamination can occur when a small number of cells are accidentally transferred from one culture to another (for example, if a bottle of medium is shared between two cultures). Initially, this results in a mixture, but the faster-growing cell line will rapidly overgrow and replace the other. The end result is a misidentified cell line—a cell line that no longer comes from the original, authentic material [3]. Misidentified cell lines may come from a different species, cell type, or disease state: all are important qualities that affect the validity of that cell line sample as a research model.

**Box 2. What Is Cell Line Authentication?**

In order to ensure a cell line model is consistent with expectations, three properties should be assessed:

1. **Identity (authenticity)**. Analysis of genotype through comparison of DNA profiles is used to establish the original source of a cell line. If the profile does not correspond to the original source material, the cell line is misidentified. In cases where the DNA profile of the individual who donated the tissue is known, a direct comparison is sufficient. In cases where original profiles are not available, comparison to the earliest possible source of a cell line is needed.
2. **Purity (contamination)**. Contamination is commonplace. Adventitious organisms (bacteria, fungi, mycoplasma, yeast, viruses) and cross-contamination with another cell line can occur but can be detected by a variety of simple tests. Contaminants such as viruses and prions are less common and require more complex testing approaches. The need for further testing is determined by the application; for example, viral testing is needed for safety of the researcher or if a cell line is in vivo or for production of biologicals.
3. **Phenotype (characterization)**. These are the complex traits of a cell line as a result of its genotype and environment. Phenotypic traits of cell lines (such as proliferation rates and drug sensitivity) can change over time in the absence of best practices in cell culture. These traits are often unique to specific cell types and individual patient samples and thus require unique tests.

A cell line can be considered as authentic if it corresponds to its original source by comparison of DNA profiles. Authentic cell lines maintained in different laboratories can possess variable phenotypes. Variations in phenotypes do not invalidate the authenticity of the cell line, which is why careful characterization of cells should be performed before experiments are conducted. While standards for identity have begun to be defined, standards to assess contaminants and phenotypic traits have yet to be defined.

In this issue, Almeida et al. [4] discuss the importance of developing standards for authentication of cell lines, with a focus on cell line identity, to address the problem of cell line misidentification. Such a call for standards extends beyond cell lines to a range of practices to improve the reproducibility of preclinical research [5]. They outline the status of existing standards, how they contribute to improved practices, and what future standards are required to ensure that cellular models are used appropriately and with confidence. The authors are affiliated with the National Institute of Standards and Technology, whose mission is to advance measurement science, standards, and technology, but they acknowledge that this is a community problem that is dependent on quality contributions from many organisations and individuals.

Here, we consider the key features of our current research climate that contribute to this problem and how standards are an important factor to help promote positive change.

## Human Nature

Natural selection favours those who cheat, resulting in an innate tendency to maximize one’s individual fitness to gain advantage. This biological hardwiring—the “selfish gene”—is evident in the behaviour of children and some adults throughout our society. To form an orderly society, we create laws that regulate certain actions to promote safety and ensure individual rights. Even in science, where empirical evidence, objectivity, and integrity are fundamental tenets, there are many examples of individuals engaging in fraudulent behaviour. This and other forms of misconduct are obvious causes of unreliable or irreproducible research, which undermines our discipline [6].

Although fraudulent behaviour is an important concern, there are more pervasive reasons why scientists continue to use invalid models. Generally, when something is not required, it does not get done. When conducting cell-based experiments, why perform an additional test to authenticate a cell line when the results support the hypothesis and the test is not required for funding or publication? If granting organisations, institutions, and journals were to require authentication to receive funding, retain research space, or publish, it is likely more researchers would adopt this practice. Several organisations now encourage or mandate authentication, but this represents only a handful of stakeholders. So why do the majority of stakeholders not follow suit?

As Almeida et al. point out, in order to require authentication, there must first be standards to serve as guidelines [4]. Stakeholders cannot require authentication if they do not know what this involves. A defined test using quality reagents and protocols to allow universal interpretation of the test results is needed, along with material standards and reference data with which to compare results. Fortunately, these exist for a relatively inexpensive authentication test for human cell lines—the Short Tandem Repeat (STR) profiling assay (Box 3). So why, for the benefit of science as a whole, do we not adopt these testing standards in a universal fashion?

**Box 3. What Is Short Tandem Repeat (STR) Profiling?**

Extensive work has been done to show that STR profiling is suitable for human identification, which the cell authentication community has used as the foundation for its work. STR profiling is based on regions of DNA that contain short repeated sequences. Testing involves PCR amplification of multiple STR loci, followed by analysis of the amplified fragments to determine the number of repeats present at each locus. These numbers can be recorded in database format, allowing scientists to compare results between laboratories. STR loci can vary extensively between individuals across the population. This means that, depending on the number of STR loci examined, the scientist can have a high degree of confidence that the STR profile is unique to that individual. STR profiling of cell lines allows scientists to compare their cell line sample to tissue or DNA from the original donor or to other cell lines to determine whether their sample is authentic or misidentified.

## Sociological Pressures: Time and Publishing

Society as a whole equates success with visible achievement, resulting in relentless stress and pressure to produce tangible results. As a result, the academic scientific community spends much of its time searching for scarce resources that often require publications to secure. Pressure to publish and to be seen to generate “novel” findings can encourage behaviour that is inconsistent with fundamental tenets of good science. Hence, it is no surprise that many researchers feel that they do not have sufficient time to assess the quality of their reagents using methods such as cell line authentication [7].

There is a pernicious belief that the quality of scientific work is directly proportional to the quality of the journal in which it is published. This has resulted in a perverse reliance on journal impact factors when assessing career success [8], a fetishizing of positive results by authors and journals [9], and a dearth of papers independently replicating prior results. Data show there is no correlation, or even an inverse association, between the quality and reproducibility of published data and impact factor [10,11]. Forty percent of landmark papers leading to Nobel Prizes were published in “lower tier” journals [12]; top journals have the highest retraction indexes [13]. While the latter may be a positive finding—correction of the literature is part of good science—it shows that scientists publishing in top journals are not immune to error. Refutation or retraction rates are increasing, often with tangible human cost [14,15].

The peer-review system has evolved to prevent poor or fraudulent science from being widely distributed. It is designed not only to gauge the merits of new scientific discoveries but also to vet the quality of the science conducted and the validity of the data. However, there are significant limitations to what a reviewer can infer from words and images on paper. Much is left to the belief in the integrity of individuals, and there are no written consensus standards for reviewers to follow. Serial protagonists of scientific fraud are often caught not during scientific review but through post-publication analysis.

Although the research organisation supports and enables the scientist’s work, the organisation is often missing when it comes to publication. Looking at high-profile cases of deliberate misconduct and retractions because of inadequate scientific rigour, serious questions should be raised regarding the degree of oversight by the fraudster’s mentors, colleagues, and institution. Good institutional practice is needed to improve scientific integrity [16]. There is also an apparent disinterest by some journals in announcing retractions in a clear and timely manner as well as imposing financial penalties for authors wishing to publish corrections to the literature [17]. For those journal editors committed to the retraction process, there are many obstacles to overcome [18]. Clearly, a wholesale rethink is needed on how we create incentives, reward good science, and present and review our findings in an objective and consistent way.

As Almeida et al. point out, defining standards and publishing the highly qualified data which drives those standards will be an integral part of this process, particularly for transparent reporting and evaluation of experimental reagents [4]. This process should be community-driven, funded, and published.

## Funding and Costs

Funding is tight and tied to publications. The typical funding agency calculates funding levels based on personnel costs, consumables, and overheads. Commonly, there is no mention of, or budget allocated for, careful assessment of the quality of reagents. To date, only the Prostate Cancer Foundation (PCF) requires adherence to best practices regarding cell line authentication from its funded investigators. The National Institutes of Health (NIH) have recently adopted a policy on rigor and transparency, requiring applicants to describe methods to ensure the identity of key biological resources such as cell lines. While a welcome step forward, the policy stops short at requiring proof of authentication. Until all funding agencies put in place similar requirements to that of the PCF and support those requirements with specific funding, only small increases will occur in the proportion of labs that perform authentication.

Cost is widely seen as a problem for cell authentication, partly because the alternative (doing nothing) is always cheaper. It may surprise some scientists to know that the costs of either STR- or SNP-based authentication are quite low, in comparison to a small aliquot of antibody or chemical reagent. Admittedly, costs can grow dramatically if large panels of cell lines are used. However, there is a general lack of understanding as to how frequently cell lines need testing, making it difficult to predict overall costs. Standards and guidelines help to clarify what is necessary and sufficient for authentication, enabling broader compliance.

## Education and Training

**Fig 1:**
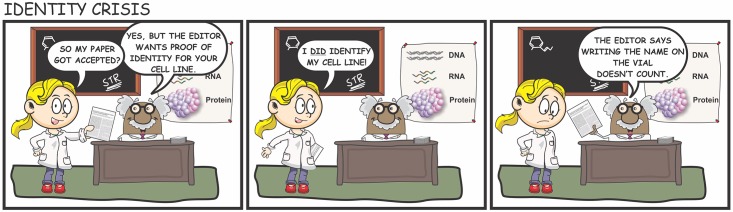
Improved training and awareness are needed to promote the use of cell line authentication. Illustration conception: ACD; artwork: RMN.

The current education system teaches scientific facts and theorem from textbooks, with an emphasis on undergraduate training. Postgraduate practical training rests on an ad hoc system of mentor- or peer- based training. Specialised cell culture textbooks and protocols are available, and online written and video protocols are becoming more widely available. However, practical knowledge is mostly learned on the job and is thus dependent on the skill and knowledge of the instructor, which can vary widely from one laboratory to the next. Training is rarely supported by funding or common resources that can be used in many different laboratories. Without a common set of standards to define best practices in cell culture, it is inevitable that individuals will receive inconsistent tutorage and pearls of ignorance will be propagated (Fig 1). To elicit real change, scientific curricula and textbooks need to include the benefits and pitfalls of the models and reagents we use, and the major stakeholders in our community must commit to defining standards, raising awareness, providing funding, and conducting training on the need for authentication and best practices in cell culture.

## Future Perspectives

There remain significant barriers to exhaustive adoption of authentication. As is the case for all research, it is difficult to guard against all misconduct; if all that is required from investigators is a table of STR profiles or copies of electropherograms, these can easily be duplicated from publicly available resources by unscrupulous individuals. Requirement of a certificate of authenticity from a certified testing facility would unfairly penalise organisations that have the necessary expertise to perform SNP or other genome-based comparisons and conduct their own internal tests. Journals could require submission of DNA to an independent body for authentication as a prerequisite for publication, but this would increase publication costs, burden the process, and would be open to potential fraud. So we are once again back to reliance on the integrity of the individual scientist.

There is hope when we look at the increasing number of stakeholders that require authentication. Early training of scientists will add to that number, as will adoption of new practices to address some fundamental flaws. We can learn from industry to place a greater emphasis on focused, good science rather than publications or big science. The need for investment down the drug development pipeline is of sufficient magnitude that mistakes or misconduct cannot be tolerated. Our goal should be for scientists to view authentication as essential and burdensome as running a molecular weight ladder on their western blots.

All the aspirations outlined here are mere fantasy without a set of community-driven standards that can define and equalise the process. Stakeholders in cell line authentication need these standards for a consistent and shared approach to improve quality. In this issue, Almeida et al. describe how this has been accomplished for STR authentication of human cell lines and comprehensively illustrate the benefits of defined standards [4]. They also describe the limitations of current standards and areas where there is still a need to develop new standards; most significantly, we lack standards for profiling nonhuman cell lines and testing other genetic or phenotypic traits required for cell line characterisation (Box 2).

Clearly, our community has a long way to go. It is now up to investigators, funding agencies, journals, and public and private enterprises to insist on the highest standards of transparency and rigor in biomedical research and to provide funding to promote research in these areas.

## Abbreviations

CC: cross-contaminated
MI: misidentified
NIH: National Institutes of Health
PCF: Prostate Cancer Foundation
STR: Short Tandem Repeat
